# Measuring blood cell DNA damage using the *PIG-A* mutation and CBMN assay in pancreatic cancer patients: a pilot study

**DOI:** 10.1093/mutage/gead006

**Published:** 2023-04-03

**Authors:** Lucy Nichols, Rachel Lawrence, Hasan Haboubi, Bilal Al-Sarireh, Shareen Doak, Gareth Jenkins

**Affiliations:** Swansea University Medical School, Swansea University, Singleton Park, Swansea, United Kingdom; Swansea University Medical School, Swansea University, Singleton Park, Swansea, United Kingdom; Swansea University Medical School, Swansea University, Singleton Park, Swansea, United Kingdom; Department of Pancreato-biliary surgery, Morriston Hospital, Swansea, United Kingdom; Swansea University Medical School, Swansea University, Singleton Park, Swansea, United Kingdom; Swansea University Medical School, Swansea University, Singleton Park, Swansea, United Kingdom

**Keywords:** biomarker, detections, pancreatic, cancer

## Abstract

Pancreatic cancer still has one of the worst prognoses of all solid malignancies, despite developments in cancer knowledge and care. Research into pancreatic cancer has not fully translated into clinical improvements and as a result, fewer than 1% of patients survive 10 years post-diagnosis. This bleak outlook for patients could be improved by earlier diagnosis. The human erythrocyte phosphatidylinositol glycan class A (*PIG-A*) assay monitors the mutation status of the X-linked *PIG-A* gene by measuring glycosyl phosphatidylinositol (GPI)-anchored proteins on the extracellular surface. We have previously identified an elevated *PIG-A* mutant frequency in oesophageal adenocarcinoma patients and here investigate whether this could be seen in a pancreatic cancer cohort, given the urgent need for novel pancreatic cancer biomarkers. In our pilot study, an elevated *PIG-A* mutant frequency (5.775 × 10^−6^ (95% CI 4.777–10) mutants per million) was seen in pancreatic cancer patients (*n* = 30) when compared to the non-cancer control group (*n* = 14) who had an erythrocyte mutant frequency of 4.211 × 10^−6^ (95% CI 1.39–5.16) mutants per million (*p* = 0.0052). A cut-off value of 4.7 mutants per million provided an AUROC of 0.7595 with a sensitivity of 70% and specificity of 78.57%. A secondary measure of DNA damage in an alternative blood cell population also showed an increase in peripheral lymphocytes using the cytokinesis-block micronucleus assay (*p* = 0.0164) (AUROC = 0.77, sensitivity = 72.22%, specificity = 72.73%). The micronucleus frequency and *PIG-A* status show some potential as blood-based biomarkers of pancreatic cancer, but further investigations of these DNA damage tests are required to assess their utility in pancreatic cancer diagnosis.

## Introduction

Despite improvements in overall cancer survival rates, pancreatic cancer (PC) survival remains largely unchanged since the 1970s. According to IARC’s Global cancer observatory, the five-year survival rate for PC is 7.93% in the UK, comparatively lower than in countries with similar economic/demographic profiles [1]. This bleak prognosis is largely attributed to late diagnosis. The majority of PC patients are diagnosed with advanced disease (stages III or IV), where curative treatment is not possible [2]. Several diagnostic challenges arise with PC. For example, imaging modalities alone struggle to differentiate between benign and malignant diseases. Endoscopic techniques for local visualisation and biopsy are invasive and have associated risks (perforation and iatrogenic pancreatitis). Final diagnoses can often only be from histological assessment of the tumour, an invasive procedure [3].

Blood-based biomarkers are less invasive and allow the repeat sampling of individuals to monitor disease progression. Currently, serum CA19-9 is the only approved blood-based biomarker for PC. However, CA19-9 cannot be a reliable indicator of pancreatic malignancy in all patients as it is a modified Lewis antigen [4] and 5–10% of the population are Lewis negative [5]. Benign disease (jaundice, pancreatitis, chronic liver disease) can also elevate CA19-9 levels [6]. The search for novel PC biomarkers is therefore of great importance to improve diagnosis and increase overall patient survival.

**Figure 1.**
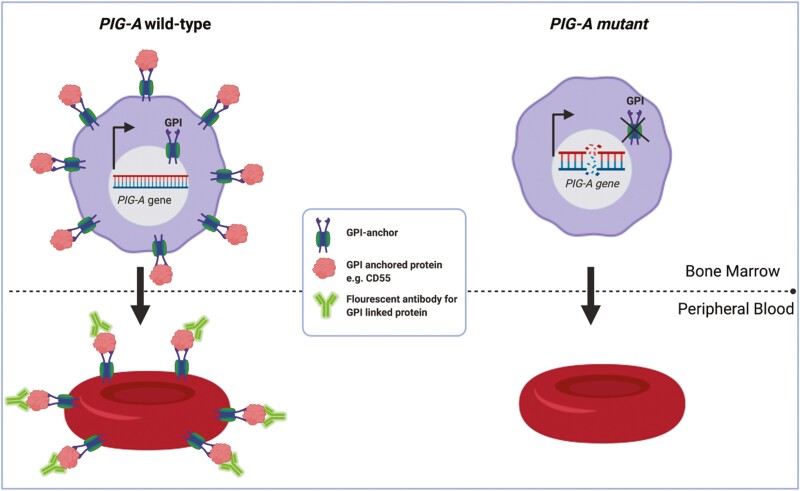
Functional phosphatidylinositol glycan anchor biosynthesis class A (*PIG-A*) gene encodes the catalytic domain of an enzyme required for glycosylphosphatidylinositol (GPI) anchor biosynthesis, identified using fluorescent antibodies targeting GPI-anchored extracellular proteins, for example, CD55. *PIG-A* mutant cell lacks both GPI anchors and GPI-anchored proteins and are non-fluorescent.

The X-linked phosphatidylinositol biosynthesis class A (*PIG-A*) gene encodes the catalytic domain of the N-acetylglucosamine transferase enzyme. This catalyses the first step in glycophosphatidylinositol (GPI) anchor biosynthesis [7]. Single, silencing mutations within *PIG-A* inactivate the enzyme, preventing GPI anchor biosynthesis [8–10]. As a result, *PIG-A* can act as a reporter gene for mutation, through fluorescent detection of GPI-anchored extracellular proteins using flow cytometry (Fig. 1).

The *Pig-a/PIG-A* assay was originally harnessed for rodent genotoxicity studies in erythrocyte and reticulocyte cell populations [11]. The human erythrocyte *PIG-A* assay quantifies GPI anchor deficiency by measuring the GPI ­anchored ­proteins CD55 and CD59 on the extracellular surface. Healthy population studies in humans have consistently reported red blood cell (RBC) *PIG-A* mutation frequencies of <5 mutant cells per million (range 2.8–4.38 × 10^−6^ mutants/million) [12–15]. Previous work by Araten *et al.* measured *PIG-A* mutant frequency *ex vivo* in lymphoblastoid cell lines and found that the mutation rate in cells isolated from patients with Fanconi anaemia was 30 times higher than that of healthy controls [16].

Given the well-established links between DNA damage and cancer, the assay has also been used in a small number of cancer studies. One study monitored the *PIG-A* mutation frequency across a wide range of malignancies in patients undergoing chemo and radiotherapy treatment [9]. Within our own research group, the assay has been used to study a cohort of patients with gastro-oesophageal reflux disease, Barrett’s metaplasia, and oesophageal adenocarcinoma where a 3.48-fold change in mutant frequency was viewed between healthy donors and those with oesophageal adenocarcinoma [13]. To date, erythrocyte *PIG-A* mutant frequency has been measured in only three patients with PC in the above-mentioned chemo-radiotherapy paper where no pre-treatment mutant frequencies were recorded [9].

Additionally, the human lymphocyte cytokinesis-block micronucleus (CBMN) assay was carried out in parallel to provide a secondary measure of DNA damage in an alternative blood cell population. The lymphocyte CBMN assay was investigated in the HUMN collaborative project, which analysed over 7000 subjects and found that elevated micronucleus frequency is associated with a higher risk of certain cancers including urogenital and gastrointestinal malignancies [17, 18].

Furthermore, given that cancer is impacted by extrinsic factors [19], and there is a strong influence of lifestyle and diet on the risk of PC development, induction of *Pig-a* mutation was studied *in vitro* following exposure to the bile acid deoxycholic acid (DCA) and the alcohol metabolite acetaldehyde. Bile acids including DCA have been shown to be carcinogenic in multiple tumour types including PC [20]. Research has demonstrated that PC patients have elevated levels of unconjugated bile acids such as DCA in the common bile duct [21] and plasma [22] when compared to controls, potentially exposing circulating blood cells to this DNA-damaging agent. This DCA-mediated carcinogenesis may be driven by the upregulation of oncogenic MUC4 expression [22]. In addition, alcohol has been implicated in PC. Risk of developing PC can be increased 1.6-fold in those who consume more than six alcoholic drinks a day [23] and a dose–response relationship exists between alcohol consumption and PC risk in a population of heavy drinkers [24]. In addition, acetaldehyde has also been shown to induce DNA damage in haematopoietic stem cells [25] that could potentially manifest as *PIG-A* mutant erythrocytes. The mouse lymphoma cell line L5178Y was used for *in vitro Pig-a* analyses because even though it is not directly related to pancreatic cancer, it is well established in genotoxicity testing and has previously been optimised for the *Pig-a* assay [26].

## Materials and methods

### Ethical approval

Research ethics committee approval was awarded for this study (REC reference: 17/SW/0012). Diagnosis of pancreatic disease was made by a pathologist from biopsies obtained during an endoscopic ultrasound procedure or explorative laparoscopic surgery. Valid informed consent was obtained from all participants. Ten millilitres of whole blood was collected by venepuncture into a lithium heparin tube (Greiner-Bio One) for analysis.

### The human erythrocyte PIG-A assay

The human erythrocyte *PIG-A* assay was carried out as described previously [13]. In short, 10 µl of whole blood was stained in triplicate with 5 µl APC anti-CD235a (diluted 1:10) (BD Biosciences cat no 551336) (0.1 µl/10^6^ cells), 20 µl PE anti-CD59 and 20 µl PE anti-CD55 (both BD Biosciences) (0.4 µl/10^6^ cells). Samples were incubated at room temperature, and protected from light for 30 min. Samples were centrifuged at 500*g* at room temperature for 5 min. Erythrocytes were washed twice in 2 ml antibody wash buffer (0.2% BSA (Sigma) in phosphate buffer saline (PBS) (Gibco)). Cell pellets were resuspended in 1 ml antibody wash buffer and transferred to flow cytometry tubes for immediate flow cytometry analysis.

Gates were drawn with respect to an unstained instrument calibration standard. CD235a is an erythrocyte-specific marker and the anti-CD235a antibody was used to isolate erythrocytes from other cell types. Cells positive for CD235a and CD55/CD59 were determined to be *PIG-A* wildtype. CD235a positive, CD55/59 negative events were determined to be *PIG-A* mutant erythrocytes. A minimum of one million wild-type erythrocytes were counted and mutant frequency was quantified as the number of mutant events per million wildtype erythrocytes.

### Lymphocyte CBMN assay

The lymphocyte CBMN assay was carried out as described previously [27]. An equal volume of heparinised whole blood was gently layered on top of Histopaque®-1077 (Sigma) and centrifuged at 400*g* for 30 min with the break and acceleration turned off. The buffy layer was isolated and washed three times in 10 ml PBS (Gibco) and was resuspended in 10 ml complete lymphocyte media (RPMI 1640 supplemented with 10% FBS, 1% glutamine, and 1% sodium pyruvate (All Gibco)) at a concentration of 1 × 10^6^ cells/ml. After 24 h, 10 µl phytohemagglutinin (PHA) (Gibco) was added to induce cell division in the lymphocytes. After a further 48 h incubation, 4.5 µg/ml cytochalasin B (Merck) was added. After a further 24 h, cells were harvested and washed three times in PBS and resuspended in an appropriate volume of PBS. One hundred microlitres of cell suspension were spun onto microscope slide using cytospin centrifugation. Slides were airdried and fixed in 90% ice-cold methanol (Merck) for 10 min. Slides were stained with 20% Giemsa (Merck).

Slides were manually scored under a light microscope and 1000 binucleated lymphocytes were examined per slide and those with micronuclei, defined by Fenech were quantified [28]. Slides were labelled with anonymous patient IDs.

### *In vitro Pig-a* assay

L5178Y cells were revived from liquid nitrogen storage and grown in complete media as previously described [26]. On day 1, cells were seeded at a density of 6 × 10^6^ cells/ml. On day 2, cells were exposed to test chemicals for 4 h: deoxycholic acid (DCA) (CAS No 302-95-4, Merck) was diluted in ethanol at doses of 20 µM and 150 µM. The doses of DCA were chosen based on previous studies measuring the effect of DCA treatment on DNA damage *in vitro* [29]. Acetaldehyde solution (CAS no 75-07-0, Merck) was diluted in full media at doses of 0.25 µM and 1 µM. Doses of acetaldehyde were taken from the publication by Kayani and Parry [30]. Following 4 h exposure, cells were washed twice and resuspended in complete media.

The *in vitro Pig-a* assay was carried out on days 1, 5, 9, 12, 15, 18, and 21 following test chemical exposure and the assay was carried out as previously described by our group [13]. Based on this time course experiment, the maximum expression period was determined to be 9 days following exposure and this time point used for the data presented here. Briefly, 2 × 10^6^ cells were added to a centrifuge tube and washed once in PBS. A dead cell control was created by heating an aliquot of cells to 90°C for 3 min. Pellets were resuspended in 200 µl Zombie Violet Fixable Viability dye (Biolegend) (1:200 dilution in PBS) and incubated in the dark for 30 min at room temperature. Cells were washed once in antibody wash buffer (0.2% BSA in PBS) before resuspending the pellet in 300 µl antibody solution containing CD45 APC (Biolegend) and CD90.2 PE (Biolegend). After incubation, cells were washed in antibody wash buffer and analysed immediately by flow cytometry.

Samples were analysed using the Beckman Coulter Navios flow cytometer and Navios software. A low flow rate was used to capture 250,000 events per sample to analyse *Pig-a* mutant events. Live and dead cells were discriminated using the zombie violet viability dye. All live cells were gated further dependent on APC-CD-45 fluorescence. APC-CD-45 positive cells were then interrogated for CD90.2 status. Mutant cells were identified as live cells, CD45 positive and CD90.2 negative.

### In vitro micronucleus test

L5178Y cells were seeded at a density of 2 × 10^5^cells ml^−1^. Twenty-four hours after seeding, cells were treated with DCA (0 µM, 20 µM, and 150 µM) and acetaldehyde (0 µM, 0.25 µM, and 1 µM) in reduced serum (5%) media. Cells were exposed for 4 h followed by a 24-h recovery period. Post recovery, cells were harvested and mononucleate cells interrogated for micronuclei using the semi-automated the Metafer (Meta Systems). Although analysing mononucleate cells, MN was still defined by the information published previously [28]

### Statistics

Due to the limited number of publications investigating erythrocyte *PIG-A* status in humans, no power calculation could be used to inform us of an appropriate sample size. RBC mutant frequencies were measured in triplicate for each participant and mean values were reported. Biological and technical replicates were carried out for the *in vitro* study. IBM SPSS version 26 was used for all statistical analyses. We tested for normality using the Kolmogorov–Smirnov goodness of fit test and determined non-parametric tests the most appropriate for our data set. Mann–Whitney *U* tests were used to test for differences between groups. Median values for each group are reported, and statistical significance was set at *P* < 0.05.

## Results

### Human erythrocyte PIG-A

**Figure 2.**
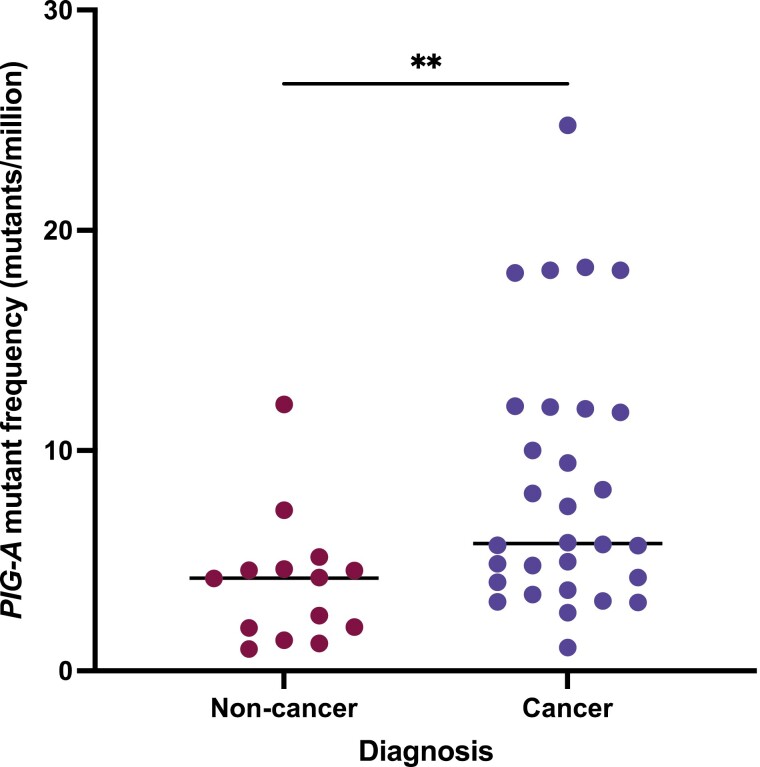
Pancreatic cancer patients (*n* = 30) had higher levels of phosphatidylinositol glycan class A (*PIG-A*) mutant erythrocytes compared to the non-cancer control group (*n* = 14) (*P* = 0.0052).

Patients with pancreatic cancer (*n* = 30) had higher levels of *PIG-A* mutant red blood cells compared to the control group which consisted of patients with benign pancreatic disease and healthy volunteers (*n* = 14) (*P* = 0.0052) as shown in Fig. 2. PC patients had an average mutant frequency of 5.775 (95% CI 4.777–10) mutants per million, compared to 4.211 (95% CI 1.39–5.15) mutants per million in the control group. Healthy and benign pancreatic donors have comparable mutation frequencies with values of 4.56 (95% CI 0.99–7.3) mutants and 3.47 (95% CI 1.947–11.97) mutants, respectively. This is similar to values seen within the literature for healthy individuals [12]. No statistical increase in mutant frequency was observed with advanced disease including tumour size (T *P* = 0.472), nodal involvement (N *P* = 0.700) and metastasis (*P* = 0.2443). In this limited cohort, whilst the cancer group were significantly older than those with benign disease (*P* = 0.002) there was no association between age and *PIG-A* mutant erythrocytes (*P* = 0.3024).

### Human lymphocyte CBMN assay

**Figure 3.**
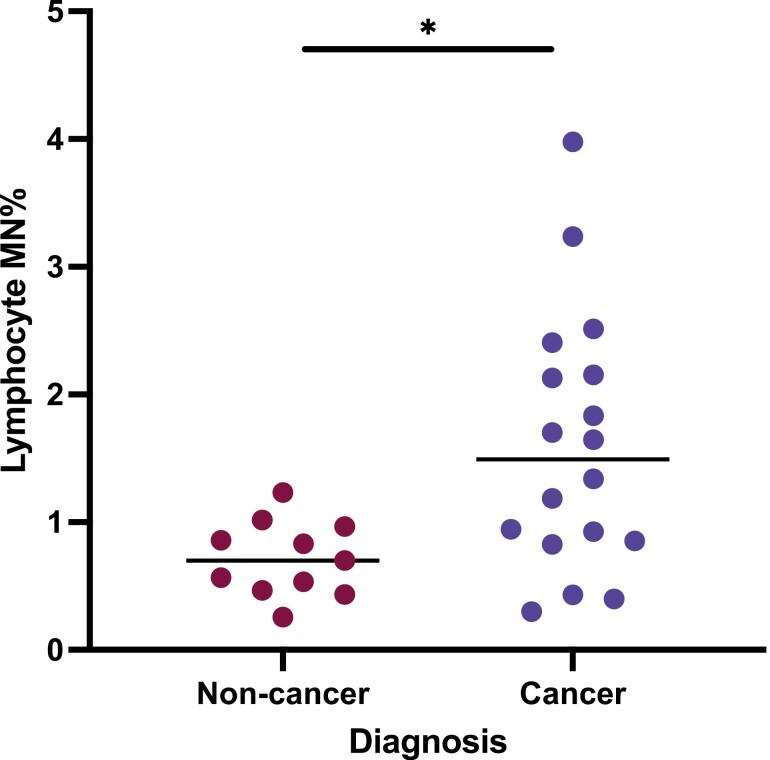
Lymphocyte micronucleus frequencies (MN%) for non-cancer controls (*n* = 11) and pancreatic cancer (PC) patients (*n* = 18). Those with PC had higher levels of MN% compared to the non-cancer group (*P* = 0.0164).

The cytokinesis-block lymphocyte micronucleus assay was carried out on 18 PC patients and 11 non-cancer controls. An elevated level of micronucleated lymphocytes was seen in PC patients with a median MN% of 1.493% (95% CI 0.8539–2.152) compared to non-cancer controls who had a median MN% of 0.7% (95% CI 0.433–1.016) (*P* = 0.0164) (Fig. 3). No difference was observed when tumour staging was explored (data not shown). There was no significant difference in age between the cancer and control groups (*P* = 0.4769) and no association between gender and MN% (*P* = 0.7992).

**Figure 4.**
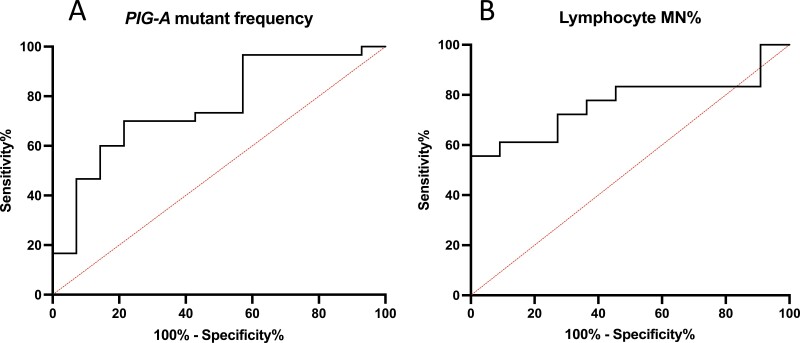
Reiver operating characteristic (ROC) curves for phosphatidylinositol glycan class A (*PIG-A*) mutant frequency and lymphocyte micronucleus frequency (MN%). A cut-off *PIG-A* mutant frequency of 4.7 mutants per million gives a sensitivity of 70% and specificity of 78.57%. A cut-off MN% of 0.8926% gives a sensitivity of 72.225 and specificity of 72.73%.

To test the ability of these two biomarkers to distinguish benign and malignant disease, receiver operating characteristic (ROC) curves were generated. For the erythrocyte *PIG-A* mutant assay, ROC analysis revealed that a cut-off score of 4.7 mutants per million would provide a sensitivity of 70% and a specificity of 78.57%, with an area under the ROC curve (AUROC) of 0.7595 (*P* = 0.006) (Fig. 4a). For the micronucleus test, a micronucleus frequency above 0.8926% provided a sensitivity of 72.22% and specificity of 72.73% with an AUROC of 0.7677 (*P* = 0.0172)(Fig. 4b).

**Figure 5.**
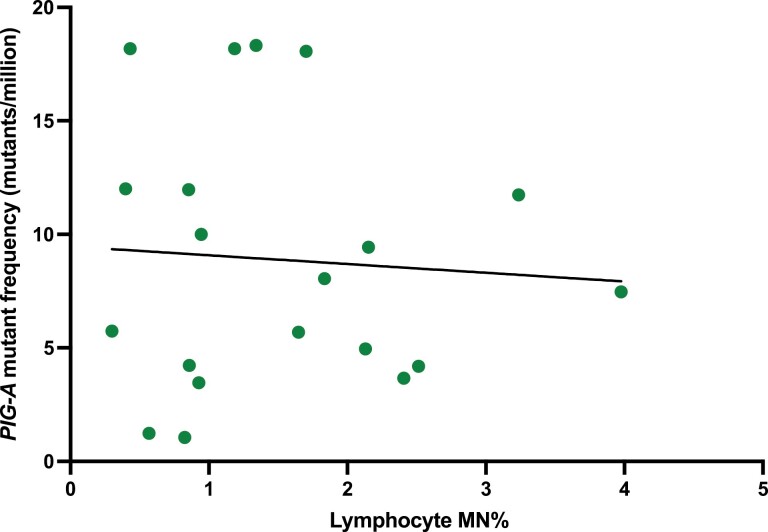
No correlation was observed between phosphatidylinositol glycan class A (*PIG-A*) mutant frequency and lymphocyte micronucleus frequency (MN%). The equation of line of best fit is *y* = −0.3856*x* + 9.465.

Next, we tested whether there was a correlation between these two DNA damage tests. Data on *PIG-A* mutant frequency and MN% was available for 20 participants. As seen in Fig. 5, there was no correlation between these two events, a higher *PIG-A* mutant frequency did not necessarily correlate with higher MN% and vice versa.

### *In vitro* studies

**Figure 6.**
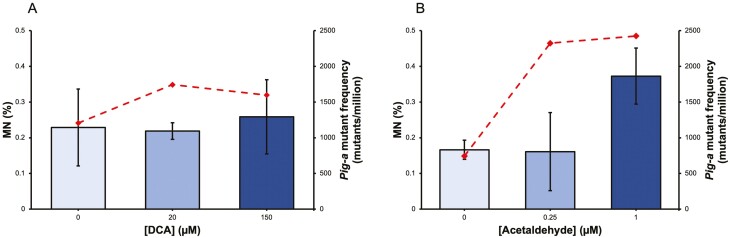
*In vitro* analysis of micronucleus frequency (MN%) and phosphatidylinositol glycan class A (*Pig-A*) mutant frequency (mutants/million) in the L5178Y cell line. Treatment with deoxycholic acid (DCA) did not increase MN% bars but caused an induction of *Pig-A* mutant cells (dashed line) at 20µM and 150 µM (*P* = 0.003). *Pig-A* mutant frequency was assessed 9 days following exposure to the test chemical. Treatment with acetaldehyde increased MN% at 1 µM and increased *Pig-A* mutant frequency (dashed line) at both 0.25 µM and 1 µM (*P* = 0.023).

To further explore the results seen *in vivo*, *in vitro* experiments were carried out. The bile acid deoxycholic acid (DCA) and the alcohol metabolite acetaldehyde were used *in vitro* to model high-fat diets and high alcohol intake, respectively. Induction of DNA damage and mutation was assessed by both the *in vitro* CBMN assay and *Pig-a* assay in L5178Y cells. DCA exposure did not induce a notable induction of MN but did increase *Pig-a* mutant frequency 9 days following exposure. Acetaldehyde exposure resulted in significant induction of MN (*P* = 0.003) at 1 µM and *Pig-a* mutant frequency (*P* = 0.023) at 0.25 µM and 1 µM concentration (Fig. 6).

## Discussion

In this study, we explored the human erythrocyte *PIG-A* assay in a cohort of patients with PC, patients with benign pancreatic disease and healthy donors. An elevated *PIG-A* mutant frequency was seen in PC patients in comparison to those with benign pancreatic disease. Further exploration of tumour staging showed a slight increase in mutant frequency in donors with metastatic PC in comparison to localised PC although this was not statistically significant. The human lymphocyte CBMN assay also showed an increased frequency of MN in donors with PC in comparison to healthy donors. Finally, *in vitro* experiments showed DNA damage induction through the mononucleate MN assay and the *in vitro Pig-a* assay in L5178Y cells following exposure to DCA, and acetaldehyde as high-fat diets and high alcohol intake respectively may contribute towards pancreatic carcinogenesis.

The median *PIG-A* mutant frequency of the non-cancer group was 4.211 × 10^−6^ (95% CI 1.39–5.16), similar to previously reported values from healthy population studies [12, 14, 15]. In this limited cohort, the *PIG-A* mutation test may be able to distinguish between benign pancreatic disease and malignant pancreatic cancer. Alternative DNA damage studies in pancreatic patients have shown conflicting data. For example, the comet assay has been explored, with a rodent necrotising pancreatitis showing no induction of DNA damage in a peripheral lymphocytes [31] but a further human lymphocyte comet assay showed a significant induction of damage, associated with disease severity in pancreatic patients [32]. A larger study across 16 laboratories found that the leukocyte comet assay is a cancer predictive biomarker and elevated DNA damage levels measured using this method are associated with overall morality [33]. Alternate methods of quantification of DNA damage and DNA mutation may have different sensitivities, for example, the comet assay may be more sensitive than the *PIG-A* mutation assay, picking up transient DNA strand breaks induced in repair processes and damage accumulated through normal physiological conditions.

Studying circulating mutational burden may indicate a disease state elsewhere in the body. In this study, an elevated *PIG-A* mutant frequency was viewed in PC patients in comparison to non-cancer controls. Previous studies within our research group have identified a 3.48-fold increase in circulating *PIG-A* mutant erythrocytes in oesophageal adenocarcinoma patients in comparison to healthy controls and patients with reflux disease [13]. This is a greater difference than the 2.075-fold change between PC donors and healthy controls seen in our pilot study. Here, the pancreatic cancer patients had a median *PIG-A* mutant frequency of 5.775 (95% CI 4.777–10) mutants per million compared to 9.75 (95% CI 4.36–17.52) mutants per million for oesophageal adenocarcinoma patients in the above-mentioned study.

The human erythrocyte *PIG-A* assay is a simple and quick assay that can provide the mutant frequency of the donor within 2 h of blood draw. This speed would be beneficial within a clinical setting, with a quick turnaround time to help influence patient management. The assay requires only 40 µl of whole blood for analysis, meaning a finger prick volume of blood would be sufficient. Furthermore, mutant cells are quantified using flow cytometry, a technique already applied clinically to diagnose paroxysmal nocturnal haemoglobinuria (PNH).

Further studies have monitored circulating DNA damage markers within PC for potential use in diagnosis. One study of peripheral blood mononuclear cells identified reduced DNA damage, shown through quantitative PCR detection of reduced amplification, in pancreatic ductal adenocarcinoma patients in comparison to controls in mitochondrial DNA (*P* = 0.03) [34]. This contrasts our findings but suggests increased DNA repair capacity within peripheral blood mononuclear cells as a result of increased oxidative stress. Furthermore, circulating cell-free DNA may be able to diagnose early-stage PC while reflecting mutations viewed within the primary pancreatic tumour [35]. Novel tumour markers are also emerging which could be harnessed for PC diagnosis including ICAM-1 [36], aiming to improve upon current biomarkers including CA19-9.

Given the complex nature of PC, a biomarker panel is likely to be a more efficient diagnostic approach. Previous studies have indicated using further biomarkers alongside clinically accepted tumour markers, including inflammatory markers, can improve performance in comparison to lone tumour markers [36]. We aimed to determine whether the addition of DNA mutation and DNA damage markers would also improve upon current markers for PC. Given the diagnostic challenges associated with CA19-9 including non-specific elevation and serum-negative patients, combination with our DNA damage assays may improve the overall performance of CA19-9. Further exploration of this is warranted due to diagnostic challenges associated with PC and the ease and simplicity of the erythrocyte *PIG-A* assay in particular.

Within our pilot study, the human erythrocyte *PIG-A* assay has shown PC patients had an elevated frequency of *PIG-A* mutant erythrocytes in comparison to benign donors. However, it is important to consider the limitations of this study including the limited number of patients recruited and the scarcity of demographic information. Further, larger studies would be required to investigate the link between DNA damage in circulating blood cells and malignancy, shedding light on the applicability of these assays as a diagnostic tool. Previously, increased *PIG-A* mutation frequency was found to be associated with disease staging in a population of patients with oesophageal adenocarcinoma [13], yet this was not viewed with the limited number of PC patients analysed here. Further questions have also been raised about the specificity of both the MN and *PIG-A* assay as an elevated level *PIG-A* mutant frequency or MN% may indicate the presence of cancer somewhere in the body. We believe such a test may still be clinically useful where patients suspected of having cancer may undergo broader investigation including imaging or other diagnostic procedures such as endoscopy depending on symptoms and medical/family history.

Although a wide range of malignancies was explored by Horibata *et al.* (2016), limitations of the study, specifically no baseline *PIG-A* mutation records before treatment, cannot determine whether malignancies outside of the oesophagus and pancreas would also show an increased mutation frequency. A recent study by Cao *et al.* discovered an increase in erythrocyte *PIG-A* mutant frequency and MN% in patients with inflammatory bowel disease compared to healthy volunteers and azathioprine treatment was not associated with any increase in *PIG-A* mutant cells but seemed to increase MN% [37]. This supports the idea that these two DNA damage endpoints are formed via different mechanisms, and individuals have different sensitivities to each type of DNA damage. Micronuclei formation can occur through aneugenic mechanisms whereby a whole chromosome is lost to the MN, or if the MN contains chromosome fragments it is caused by double-strand breaks. Whereas the loss of the *PIG-A* phenotype can be caused by different types of mutations, although one study in PNH patients discovered that truncating mutations were the most abundant [38]. This mechanistic difference is important, as seen in this study, PC patients who do not necessarily have a high *PIG-A* mutant frequency can potentially be picked up using the MN assay.

## Conclusion

Given the bleak prognosis of most PC patients, the search for novel diagnostic tools for PC is of great importance. Both the erythrocyte *PIG-A* assay and the lymphocyte MN assay have shown possible usefulness within this pilot study, but larger studies are required to further explore these tests in different malignancies and determine the mechanisms by which these measures of DNA damage are linked to carcinogenesis.

## Data Availability

Data will be made available to other researchers post publication upon reasonable request to the corresponding author. Information on individual patients or participants will not be made available.
